# Active Surveillance of Iodinated Contrast Media-Induced Reactions and Associated Risk Factors Among Patients Undergoing Radiologic Procedures in Tertiary Hospitals in Tanzania: A Prospective Cohort Study

**DOI:** 10.7759/cureus.100654

**Published:** 2026-01-03

**Authors:** Riziki S Shemula, Manase Kilonzi, Elias Bukundi, Mathias E Mlugu, Ritah F Mutagonda

**Affiliations:** 1 Human and Veterinary Medicine Control, Tanzania Medicines and Medical Devices Authority, Dodoma, TZA; 2 Clinical Pharmacy and Pharmacology, Muhimbili University of Health and Allied Sciences, Dar es Salaam, TZA; 3 Epidemiology and Biostatistics, Muhimbili University of Health and Allied Sciences, Dar es Salaam, TZA; 4 Pharmaceutics and Pharmacy Practice, Muhimbili University of Health and Allied Sciences, Dar es Salaam, TZA

**Keywords:** adverse reactions, contrast-associated acute kidney injury, iodinated contrast media, radiation safety, radiologic procedures

## Abstract

Background: Exposure to iodinated contrast media (ICM), particularly at higher doses, carries significant risks of acute hypersensitivity and organ toxicity, especially involving the kidneys and cardiovascular system. Documenting these reactions is vital for patient safety, risk management, and medico-legal considerations. This study aimed to determine the incidence of ICM-induced adverse reactions and to identify associated risk factors among patients undergoing radiologic imaging in tertiary hospitals in Tanzania.

Materials and methods: This prospective cohort study enrolled 283 patients undergoing contrast-based radiologic procedures between March and May 2024 at two tertiary hospitals in the Ilala district, Tanzania. Data on demographics, drug history, comorbidities, and prior contrast exposure were collected through structured questionnaires and patient files. Blood pressure and BMI were measured, with hypertension defined as BP ≥140/90 mmHg or a history of treatment. Reactions were assessed at baseline and post-procedure at 24, 72, and 168 hours. Data analysis was performed using SPSS version 27 (IBM Corp. Released 2020. IBM SPSS Statistics for Windows, Version 27.0. Armonk, NY: IBM Corp.), applying univariate and multivariate analysis, with p-values <0.05 considered statistically significant.

Results: The incidence of any ICM-induced reaction was 39.2% (111/283). Acute reactions predominated, including gastrointestinal (27.9%), neurological (18.0%), and dermatological (13.5%) symptoms, while delayed events were mainly contrast-associated acute kidney injury (6.3%) and delayed cutaneous reactions (4/111, 3.6%). Significant risk factors included normal BMI (adjusted risk ratio (ARR) = 1.445), overweight (ARR = 1.305), neurological conditions (ARR = 1.496), cardiopulmonary disease (ARR = 1.335), oncologic indications (ARR = 1.350), anemia (ARR = 1.490), and stage 2 renal failure (ARR = 2.143). Interestingly, diabetes mellitus was associated with a lower risk (ARR = 0.540).

Conclusions: These findings highlight that acute reactions are more common than delayed ones, emphasizing the need for careful risk assessment and targeted preventive strategies during contrast procedures.

## Introduction

Iodinated contrast media (ICM) are widely used in radiology to enhance visualization of internal structures [1]. They are primarily administered intravenously, although intra-arterial and intrathecal routes are also employed [2]. Despite their diagnostic utility, ICMs can cause a range of adverse reactions, from mild allergic responses to severe life-threatening events [3]. The safety profile of ICMs depends on properties such as ionicity, iodine concentration, osmolarity, and viscosity. Non-ionic agents are generally preferred because their lower osmolality reduces the likelihood of hypersensitivity reactions [4].

Adverse reactions to ICM are typically classified as acute or delayed. Acute reactions, including anaphylactoid responses and contrast-associated acute kidney injury (CA-AKI), are more common with high-osmolar contrast agents, particularly in patients with pre-existing renal dysfunction [5]. CA-AKI is a significant complication caused by renal vasoconstriction, oxidative stress, and direct tubular toxicity, potentially leading to reversible or irreversible kidney damage [6]. Cardiovascular complications may range from mild hypotension to severe arrhythmias [2,3]. Delayed reactions, often immunologically mediated, can occur hours to days after ICM administration and may involve dermatological or systemic manifestations [7].

From a medico-legal perspective, healthcare providers face growing obligations related to informed consent, documentation, and risk mitigation in the administration of contrast agents. Inadequate management of ICM reactions can result in liability, underscoring the necessity of adherence to clinical guidelines and the implementation of proactive safety measures [8,9].

In Tanzania, the use of contrast-enhanced diagnostics has increased rapidly; however, there is limited research on ICM-related complications beyond nephrotoxicity. [10,11]. This study aimed to determine the incidence of ICM-induced adverse reactions and to identify associated risk factors among patients undergoing radiologic imaging in tertiary hospitals in Tanzania.

## Materials and methods

Study design and setting

A hospital-based, single-arm prospective cohort study was conducted from March to May 2024 at two tertiary care centers in Dar es Salaam: Muhimbili National Hospital (MNH) and Jakaya Kikwete Cardiac Institute (JKCI). These hospitals serve as national referral centers, receiving patients requiring advanced radiologic imaging for diagnostic and therapeutic purposes. Both institutions follow standardized radiology protocols and routinely use non-ionic low-osmolality ICM for contrast-enhanced imaging procedures.

Study participants and eligibility criteria

The study included consenting adult patients who underwent radiologic procedures involving iodinated contrast agents at either MNH or JKCI. Patients were eligible if they had no prior contraindications to ICM and could be followed up for adverse event monitoring. Patients who were unwilling/unable to provide consent or complete follow-up were excluded. Patients with documented prior severe contrast reactions were not routinely scheduled for contrast-enhanced imaging and were therefore not enrolled. Muhimbili University of Health and Allied Sciences Institutional Review Board issued approval MUHAS-REC-03-2024-2120

Sample size and sampling technique

A sample size of 283 patients was determined using Cochran’s formula, accounting for a 20% nonresponse rate, a 5% precision error, and a 1.96 confidence level [12]. The initial proportion of contrast media reactions was estimated at 19% in a study in Tanzania [11]. Participants were recruited using consecutive sampling. This approach minimized selection bias by enrolling all eligible patients in sequence as they presented for procedures.

Data collection process

Data were collected by three trained research assistants (registered nurses) and the principal investigator (a pharmacovigilance expert pharmacist). A pretested structured questionnaire covering key domains, including sociodemographic characteristics, medical history, medication use, comorbidities, prior ICM exposure, and history of medication allergy, was used. The questionnaire was informed by the literature review, expert consultation, and the investigator's experience. Pretesting was conducted among approximately 10% of the expected sample size to ensure clarity, internal consistency, and feasibility in the study setting.

Additional data, including laboratory findings (e.g., serum creatinine, hemoglobin) and clinically documented complications, were extracted from patient medical files. Participants were actively followed up at 0-60 minutes (acute phase), 24 hours, 72 hours, and 7 days post-procedure. Patients discharged before day 7 were followed up via phone. Physical assessments (height, weight, blood pressure) were conducted using standardized equipment and procedures.

BMI was categorized using WHO standards: underweight (<18.5 kg/m²), normal (18.5-24.9 kg/m²), overweight (25.0-29.9 kg/m²), and obese (≥30.0 kg/m²). All contrast-enhanced procedures utilized intravenously administered non-ionic low-osmolality ICM, with volumes typically ranging from <100 mL to 200 mL, depending on procedure type and clinical indication. Contrast agent selection was guided by institutional procurement policies and availability during the study period. Delayed adverse events among patients discharged before day 7 were assessed using a standardized phone-based checklist focusing on dermatological symptoms, renal symptoms, hospital re-attendance, and clinician-diagnosed adverse events.

Statistical analysis

Data were analyzed using SPSS Statistics version 27 (IBM Corp. Released 2020. IBM SPSS Statistics for Windows, Version 27.0. Armonk, NY: IBM Corp.). Renal function was classified using estimated glomerular filtration rate (mL/min/1.73 m²) according to KDIGO guidelines: stage 2 (60-89) and stage 3 (30-59). Descriptive statistics were summarized using frequencies and percentages.

A multi-stage analytical approach was used to identify factors associated with ICM-induced reactions. First, bivariate associations were assessed using chi-square tests. Variables with a p-value <0.20 in bivariate analysis were entered into univariate Poisson regression models. Variables meeting this threshold, together with clinically relevant covariates (e.g., age, BMI, renal function, comorbidities), were considered for inclusion in the multivariate Poisson regression model. Variables retained in the final model were selected based on statistical significance and clinical relevance, with p-values <0.05 considered significant. Adjusted risk ratios (ARRs) with 95% confidence intervals were reported to quantify independent associations.

## Results

Sociodemographic and clinical characteristics

Out of the 283 participants, 146 (51.6%) were female, and 135 (47.7%) were aged between 41 and 65 years. Based on BMI, 132 (46.6%) participants were classified as overweight. Most participants, 267 (94.3%), reported no prior history of medication-related allergic reactions, while 16 (5.7%) had a documented history of medication allergy. The majority received a contrast dose of less than 100 mL (246, 86.9%). Regarding indications for imaging, vascular and interventional procedures were the most common (85, 30.0%), followed by gastrointestinal and abdominal imaging (75, 26.5%) (Table 1).

**Table 1:** Sociodemographic and clinical characteristics of the study participants (N = 283) BMI: body mass index, NSAIDs: nonsteroidal anti-inflammatory drugs

Variable	Frequency (n)	Percent (%)
Sex		
Male	137	48.4
Female	146	51.6
Age (median = 56)		
18-40	83	29.3
41-65	135	47.7
>65	65	23.0
Residency		
Inside Dar es Salaam	157	53.4
Outside Dar es Salaam	126	46.6
BMI (median = 26.84)		
Underweight	4	1.4
Normal weight	84	29.7
Overweight	132	46.6
Obesity	63	22.3
Allergic to any medications		
Yes	16	5.7
No	267	94.3
Indication for radiologic study		
Cardiopulmonary	45	15.9
Gastrointestinal and abdominal	47	26.5
Oncological	53	18.7
Neurological	25	8.8
Vascular and procedural	85	30.0
Dose of contrast (ml) <100	246	86.9
100-200	36	12.7
>200	1	0.4
Medical history		
Heart problem	51	18.0
Thyroid disorders	2	0.7
Previous use of contrast media	20	7.1
Cigarette smoking	18	6.4
High cholesterol	3	1.1
Diabetes mellitus	17	6.0
Asthma	5	1.8
Alcohol consumption	38	13.4
Anemia	10	3.5
Hypertension		
Stage 1	21	7.4
Stage 2	29	10.2
Stage 3	19	6.7
Renal failure		
Stage 2	11	3.9
Stage 3	15	5.3
Previous medication use		
Diuretics	61	21.6
Angiotensin 2 receptor blockers	40	14.1
Calcium channel blockers	7	2.5
Angiotensin-converting enzyme inhibitor	2	2.5
NSAIDs	20	7.1
Over-the-counter/other medications	11	3.9
Clopidogrel	25	8.8
Atorvastatin	21	7.4

Incidence of ICM-induced reaction

**Figure 1:**
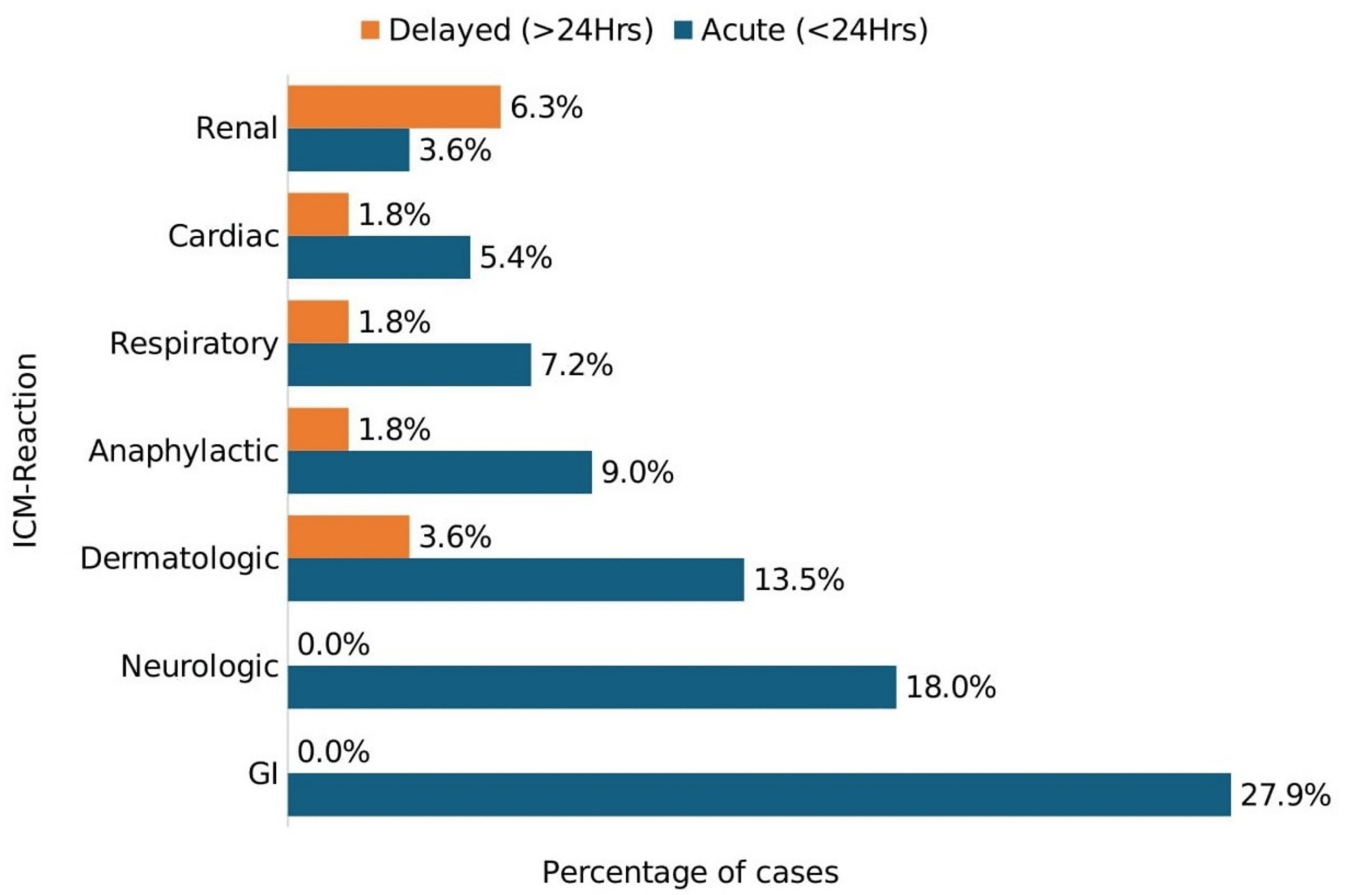
Distribution of acute and delayed ICM reactions among participants (N = 111) GI: gastrointestinal, ICM: iodinated contrast media

Overall, 111 of 283 participants (39.2%) experienced ICM-induced reactions. Figure 1 shows the distribution of acute and delayed reactions. Among acute events, gastrointestinal complications were most frequent (27.9%), followed by neurological (18.0%) and dermatological reactions (13.5%). Delayed events were less common and primarily included CA-AKI (6.3%) and delayed cutaneous reactions (3.6%).

Factors associated with ICM-induced reactions among study participants

Following multivariate analysis, the factors associated with the incidence of ICM-induced reactions were normal weight BMI (ARR = 1.45, 95% CI: 1.047-2.00, p = 0.025), overweight BMI (ARR = 1.31, 95% CI: 0.99-1.73, p = 0.043), oncological indication (ARR = 1.35, 95% CI: 1.02-1.80, p = 0.039), neurological indication (ARR = 1.50, 95% CI: 1.12-2.00, p = 0.007), stage 2 renal failure (ARR = 2.14, 95% CI: 1.21-3.81, p = 0.009), and anemia (ARR = 1.49, 95% CI: 1.14-1.95, p = 0.003). Conversely, diabetes mellitus was found to be protective (ARR = 0.54, 95% CI: 0.30-0.97, p = 0.033) (Table 2).

**Table 2:** Factors associated with ICM-induced reactions among study participants (n = 283) CRR: crude risk ratio, ARR: adjusted risk ratio, CI: confidence interval, BMI: body mass index, KDIGO: Kidney Disease: Improving Global Outcomes, ICM: iodinated contrast media

Variable	Categories	n (%)	Univariate analysis CRR (95% CI)	P-value	Multivariate analysis ARR (95% CI)	p-value
Age (years)	18-40	35 (48.6)	3.21 (1.21-8.75)	0.013	1.12 (0.71-1.77)	0.613
	41-65	54 (40.3)	1.16 (0.90-1.49)	0.161	1.16 (0.90-1.49)	0.261
	>65	22 (28.6)	Ref		Ref	
BMI (kg/m²)	Underweight	2 (50.0)	1.21 (1.08-5.91)	0.193	1.38 (0.74-2.59)	0.310
	Normal weight	43 (51.2)	0.45 (0.05-2.20)	0.040	1.45 (1.047-2.0)	0.025
	Overweight	70 (53.0)	3.15 (1.13-4.27)	0.031	1.31 (0.99-1.73)	0.043
	Obese	19 (20.2)	Ref		Ref	
Indication for radiologic procedure	Cardiopulmonary	20 (54.1)	1.06 (0.70-2.37)	0.104	1.36 (1.01-1.77)	0.045
	Gastrointestinal	30 (42.9)	1.25 (1.03-1.76)	0.159	1.25 (0.93-1.67)	0.138
	Oncological	24 (53.3)	1.20 (1.11-2.92)	0.082	1.35 (1.02-1.80)	0.039
	Neurological	13 (72.2)	2.19 (0.92-5.10)	0.013	1.50 (1.12-2.00)	0.007
	Vascular and procedural	24 (21.2)	Ref		Ref	
Contrast dose (ml)	<100	92 (37.4)	0.77 (0.28-2.74)	0.021	1.767 (0.98-3.54)	0.120
	100-200	16 (45.4)	3.43 (1.30-6.63)	0.015	2.36 (1.00-4.68)	0.055
	>200	0 (0.0)	Ref		Ref	
Hypertension	No	91 (49.2)	0.14 (1.43-3.11)	0.017	1.34 (0.84-2.15)	0.217
	Stage 0 (elevated)	5 (14.3)	1.24 (0.88-1.91)	0.151	0.97 (0.51-1.86)	0.925
	Stage 1	10 (28.6)	Ref		Ref	
Renal failure (KDIGO stage)	Stage 1	102 (40.5)	0.99 (0.72-2.11)	0.041	1.59 (0.91-2.79)	0.104
	Stage 2	5 (71.4)	1.03 (1.01-2.50)	0.109	2.14 (1.21-3.81)	0.009
	Stage 3		Ref		Ref	
Anemia	Yes	5 (100.0)	1.1 (0.95-2.23)	0.119	1.49 (1.14-1.95)	0.003
	No	96 (34.5)	Ref		Ref	
Diabetes mellitus	Yes	3 (10.0)	3.15 (1.31-6.05)	0.112	0.54 (0.31-0.95)	0.033
	No	109 (43.1)	Ref		Ref	
Alcohol use	Yes	12 (15.5)	1.02 (0.89-1.55)	0.178	1.02 (0.77-1.36)	0.878
	No	99 (41.9)	Ref		Ref	
Heart conditions	Yes	18 (24.0)	2.12 (0.91-3.14)	0.106	1.02 (0.68-1.53)	0.907
	No	93 (44.7)	Ref		Ref	

## Discussion

This study determined the incidence and risk factors of ICM-induced adverse reactions among patients undergoing diagnostic and interventional radiology in Tanzania. Overall, a high incidence of ICM-induced reactions was observed. The most common acute reactions were gastrointestinal, neurological, and dermatological complications, while delayed reactions primarily involved renal and dermatological complications. The occurrence of these reactions was statistically significantly associated with patient BMI, renal function, and selected chronic comorbidities, including hypertension, diabetes mellitus, and other underlying conditions.

Our study observed a high incidence of ICM-induced reactions (39.2%), exceeding reports from Japan (12%) and the USA (1%) [13,14], and slightly higher than estimates from African settings with comparable socioeconomic contexts (9.9-35.9%) [15-17]. This relatively high incidence should be interpreted in light of the prospective active-surveillance design, multiple follow-up time points, and broad outcome definition, which were intended to enhance detection of both mild and clinically significant events but may limit direct comparability with studies using passive surveillance or stricter definitions. Acute reactions predominated, with gastrointestinal (27.9%) and neurological (18.0%) symptoms most frequent, in line with global patterns [18,19]. Anaphylactic reactions (10.8%) were not uncommon; while some literature cites lower rates (≈0.2%) [20], such variation may reflect differences in population risk profiles, contrast formulations, case definitions, and intensity of monitoring. Delayed reactions were primarily renal (6.3%) and dermatologic (3.6%), within the global range (0.5-30%) [18,19]. Underreporting remains a concern in many sub-Saharan African settings due to limited post-procedure follow-up systems [15]. Our estimates also exceed those from Mexico (acute 26.3% and delayed 10.1%) [21], likely reflecting differences in study design and surveillance intensity; the single-arm prospective design used here facilitated real-time detection and improved event capture. These findings underscore the value of strengthened post-procedure follow-up to enhance detection and management of ICM-related reactions.

Several patient factors influenced ICM reactions. Overweight (53%) and normal-weight (51.2%) patients had higher reaction rates than obese patients (20.2%), a pattern consistent with findings from previous studies conducted in Tanzania (19). Similarly, a study conducted in Australia found that BMI is a risk factor for CA-AKI [22]. Stage 2 renal failure significantly increased the risk (RR = 2.14, p = 0.009), consistent with studies conducted in South Africa, Nigeria, and Kenya, which show that elevated creatinine levels double the risk [16,17,23]. Patients with neurological, cardiopulmonary, and oncological conditions had higher susceptibility, likely reflecting disease severity, immune compromise, and treatment-related vulnerability. The findings align with previous studies, which support that neurological risks may stem from blood-brain barrier compromise [24]. While other studies support that oncological patients had a 0.3-2.3% risk of getting ICM reactions, of which 11% develop AKI [25,26]. Anemia was also independently associated with ICM-induced reactions (RR = 1.49, p = 0.003), possibly due to impaired oxygen delivery, reduced physiological reserve, and vascular instability [27].

Although a prior history of medication allergy was not independently associated with ICM-induced reactions after adjustment, a higher proportion of reactions was observed among patients with documented drug allergies. While limited by small reported numbers, this finding remains clinically relevant and supports guideline recommendations for careful pre-procedure allergy assessment and heightened monitoring in this subgroup. In contrast with global studies linking high HbA1C (≥8.8%) with increased risk of ICM-induced reactions, our study found that participants with diabetes mellitus had a protective effect [28]. The observed association between diabetes mellitus and reduced risk of ICM-induced reactions should be interpreted cautiously. This finding is not suggestive of a protective biological effect. Still, it is more likely explained by unmeasured confounding, particularly the routine implementation of preventive measures such as hydration protocols in patients with diabetes mellitus [29,30], which were not systematically documented in this study.

Several limitations should be considered when interpreting these findings. The single-arm observational design restricts causal inference, and results should be interpreted as associative. The relatively high incidence of ICM-induced reactions may reflect the prospective active-surveillance approach, multiple follow-up time points, and inclusion of both mild and clinically significant events. Adverse reactions were not graded using international severity scales (e.g., American College of Radiology or European Society of Radiology), which may limit comparability; however, reactions were consistently classified by timing and organ system, and CA-AKI was defined using KDIGO criteria. Outcome assessment partly relied on interviews and phone follow-up, which may have introduced reporting bias, although standardized tools and trained data collectors were used. Preventive measures such as hydration protocols were not systematically documented, which may have influenced renal outcomes, including the observed protective association with diabetes mellitus. Finally, the study was conducted in two urban tertiary hospitals, which may limit generalizability beyond similar referral settings.

## Conclusions

This study demonstrates that reactions to ICM are frequent and clinically significant, particularly in tertiary hospitals in Tanzania. Acute reactions were more common than delayed events, with gastrointestinal, neurological, and dermatological symptoms being most frequently reported, while delayed reactions primarily affected renal function. Key risk factors included normal and overweight BMI, neurological, oncological, and cardiopulmonary conditions, anemia, and stage 2 renal impairment. The association observed with diabetes mellitus should be interpreted cautiously and likely reflects unmeasured preventive practices rather than a true protective effect.

These findings support the need for context-specific risk assessment protocols, improved documentation of contrast exposure and preventive measures, and structured post-procedure monitoring, particularly for patients at high risk. Future studies incorporating standardized severity grading and broader, multicenter designs are warranted to improve comparability and support the development of context-specific contrast safety guidelines.
